# Nature‐Inspired 3D Spiral Grass Structured Graphene Quantum Dots/MXene Nanohybrids with Exceptional Photothermal‐Driven Pseudo‐Capacitance Improvement

**DOI:** 10.1002/advs.202204086

**Published:** 2022-08-26

**Authors:** Peng Chang, Hui Mei, Yu Zhao, Longkai Pan, Minggang Zhang, Xiao Wang, Laifei Cheng, Litong Zhang

**Affiliations:** ^1^ Science and Technology on Thermostructural Composite Materials Laboratory School of Materials Science and Engineering Northwestern Polytechnical University Xi'an 710072 China

**Keywords:** 3D printing, GQDs/MXene nanohybrids, light‐trapping spiral grass structure, photothermal supercapacitors, structure‐enhanced photothermal‐driven capacitance enhancement

## Abstract

Solar‐thermal conversion is considered as a green and simple means to improve the performance of energy storage materials, but often limited by the intrinsic photothermal properties of materials and crude structure design. Herein, inspired by the unique light trapping effect of wide leaf spiral grass during photosynthesis, a biomimetic structural photothermal energy storage system is developed, to further promote the solar thermal‐driven pseudo capacitance improvement. In this system, three‐dimensional printed tortional Kelvin cell arrays structure with interesting light trapping property functions as “spiral leaf blades” to improve the efficiency of light absorption, while graphene quantum dots/MXene nanohybrids with wide photothermal response range and strong electrochemical activity serve as “chloroplast” for photothermal conversion and energy storage. As expected, the biomimetic structure‐enhanced photothermal supercapacitor achieves an ideal solar thermal‐driven pseudo capacitance enhancement (up to 304%), an ultrahigh areal capacitance of 10.47 F cm^−2^, remarkable photothermal response (surface temperature change of 50.1 °C), excellent energy density (1.18 mWh cm^−2^) and cycling stability (10000 cycles). This work not only offers a novel enhancement strategy for photothermal applications, but also inspires new structure designs for multifunctional energy storage and conversion devices.

## Graphical abstract

A biomimetic structural graphene quantum dots/MXene photothermal supercapacitor inspired by the light‐trapping effect of wide‐leaf spiral grass is developed. The novel bionic structural supercapacitor achieves remarkable photothermal response (surface temperature of 67.6 °C under one‐sun illumination) and record‐high solar thermal‐driven pseudo‐capacitance enhancement (up to 304% at high rate) that will open new frontiers for the application of solar thermal energy.

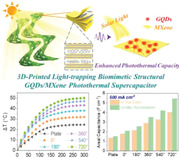

## 1 Introduction

Energy storage devices are an essential part in today's daily life because of our dependence on numerous electronic devices. As a promising energy storage device, supercapacitors (SCs) have a wide range of applications in electric vehicles, consumer electronics and sustainable energy systems, benefiting from their high power density, long cycle stability, ultrafast charging/discharging rates and excellent reliability.^[^ ^1^ ^]^ Electrode materials have been widely considered to be the main factor in deciding the electrochemical behavior of energy storage devices.^[^ ^2^ ^]^ During the past decade, there were intensive efforts to develop cheap yet powerful electrode materials. However, in this intense pursuit, the design and composition of new advanced energy materials became more and more complex, the cost got higher and higher, the synthesis process became more and more cumbersome, but the performance got closer to the ceiling, and the improvement got smaller and smaller. This is undoubtedly evolving into an arms race. Therefore, a renewable, universal, and low‐cost strategy to further boost the acquired excellent performance is highly expected.^[^ ^3^ ^]^

In recent years, the utilization of photothermal effect and microwave thermal effect to promote the conversion and production of different forms of energy has become a hot topic. For example, converting light radiation into heat energy (photothermal conversion) is a convenient and efficient method of energy harvesting. Photothermal conversion has obtained many successful applications in energy recovery, catalysis, desalination, and other fields.^[^ ^4^ ^]^ There are two definite advantages of introducing the photothermal effect into the electrochemical energy storage process. First, the photothermal electrode can realize self‐heating under solar light irradiation, which can effectively address the problems of diminishing power or even failure of SCs at lower temperatures. Besides, due to the localized heating effect, the heat is limited to the surface of the photothermal electrode without influencing the electrolyte temperature.^[^ ^4c^ ^]^ Recently, the photothermal effect of carbon‐based nanomaterials such as graphene and carbon nanotubes, has been used to enhance the electrochemical performance of SCs.^[^ ^5^ ^]^ For example, Liu et al. fabricated a three‐dimensional (3D) hierarchical graphene double‐layer‐type SC with ≈3.7 times enhancement of capacitance under 1 solar illumination.^[^ ^5a^ ^]^ Chen et al. reported that the capacitance of the photosensitive graphene/carbon nanotube hybrids electric double‐layer SC increased by 1.9 times under one solar light illumination.^[^ ^5b^ ^]^ Unfortunately, the relatively low specific capacitance and inferior energy density reported by the electric double‐layer capacitor from their intrinsic electric double‐layer mechanism severely impedes their commercialization. For pseudo‐type SCs with high theoretical capacity, the highest solar thermal‐driven capacitance enhancement reported so far is only ≈1.5 times, which is far from meeting the requirements of practical applications.^[^ ^5a^ ^]^ The further improvement of photothermal‐driven capacitance enhancement effect for pseudo‐type SCs remains a great challenge.

MXene, an emerging family of multifunctional two‐dimensional (2D) materials containing a large group of early transition metal carbides, nitrides and carbonitrides with many exceptional superiorities embracing high surface area, good conductivity, ease of functionalization, mechanical stability and great biocompatibility, has attracted great interests and showed very competitive performances in a gamut of applications ranging from energy storage, optoelectronics, wireless communication and electromagnetic interference shielding to catalysis, sensing and biomedical engineering.^[^ ^6^ ^]^ Besides the superb pseudo‐capacitance as SC electrode materials based on ion intercalation mechanism, Ti_3_C_2_T*_x_*, as the most typical representative of 2D MXene, has already shown great promise in the application of solar energy conversion due to its extraordinary light‐absorbing ability in the near‐infrared and 100% internal photothermal conversion efficiency.^[^ ^7^ ^]^ However, the application of photothermal effect from Ti_3_C_2_T*_x_* in the energy storage process has rarely been reported.

Although 2D Ti_3_C_2_T*_x_* MXene exhibits various advantages, the face‐to‐face self‐restacking of layered nanostructures and ease of surface oxidation severely restrict the accessibility of surface active sites to electrolytic ions as well as the heat exchange contact and energy storage capabilities, resulting in both underutilized capacitance and unsatisfied solar thermal conversion efficiency.^[^ ^8^ ^]^ Moreover, the inherent shortcomings of a single material also greatly limit the further improvement of its performance. To overcome the above bottleneck issues, one effective approach is introducing electro‐active nano‐carbon materials (e.g., graphene, carbon nanotubes, pyrolytic carbon) as interlayer spacers to transform 2D MXene sheets into 3D architectures with exposed surface‐facets, which has been proven effective against the re‐stacking issue and undesirable oxidation to achieve better capacity and enhanced photothermal properties.^[^ ^9^ ^]^ Specifically, zero‐dimensional (0D) graphene quantum dots (GQDs) characterized by an atomically thin graphitic plane with lateral dimensions typically <10 nm, have been widely investigated as SC and photothermal conversion materials due to their strong optical absorbance in shorter wavelengths (<1000 nm), promising photothermal conversion efficiency, a large theoretical specific capacitance and a highly functional surface.^[^ ^10^ ^]^ Inspired by these distinct and intriguing properties, it is prospective that the nanohybrids of 0D GQDs (electroactive and photo‐responsive spacers) and 2D Ti_3_C_2_T*_x_* MXene (multifunctional nanoplatforms) can convert the planar configuration of MXene into an 3D interconnected structure with open architectures and a wide absorption range, to overcome the disreputable restacking and oxidation problems, and further obtain improved photothermal and super capacitive performances. Therefore, it will be a great significance and challenge to obtain the GQDs/Ti_3_C_2_T*_x_* MXene nanohybrids.

Structure design is known to play a critical role in the overall performance of photothermal electrodes.^[^ ^11^ ^]^ In the long history of evolution, nature has created countless miracles, especially various ingenious structures from the macro world to the micro scale.^[^ ^12^ ^]^ Many plants and animals have evolved unique structures to promote their survival in special environments.^[^ ^13^ ^]^ Nature seems to have already solved all the problems and guided human beings to resolve the limitations of materials and engineering technologies, such as mechanical materials inspired by whale whiskers,^[^ ^14^ ^]^ hydrophilic materials imitated pitcher grass^[^ ^15^ ^]^ and triboelectric materials mimicked from jellyfish.^[^ ^16^ ^]^ Wide‐leaf *Albuca namaquensis* Baker (spiral grass), a type of perennial bulbous succulent plant with special spring‐like leaves, has attracted significant interest. Wide‐leaf *Albuca namaquensis* Baker likes a sunny, warm and dry environment, is resistant to drought, and is afraid of insufficient light. Interestingly, the curling of the leaves is largely dependent on light conditions. During the growth period, if the sunlight is insufficient, the leaves will be thin and weak, curled poorly, and the leaf base will be brittle, which will easily cause the leaves to fall and break. While the leaves usually curl better and keep green all the year round without withering if given good light. Hence, curling and twisting of leaves may significantly improve the efficiency of light absorption in light environments, further enhance the photosynthesis in plant chloroplast and inspire us to design high‐efficiency devices related to solar energy harvesting and conversion.

In this work, we mimicked the hierarchical structures of *Albuca namaquensis* Baker's spiral leave blades from macro to micro scale and constructed GQDs/MXene hybrid nanocoating with a wide photothermal‐response range and strong electrochemical activity (microscale “chloroplast”) on 3D‐printed torsional Kelvin cell lattice arrays (macroscale “leaf blade”) to realize solar thermal‐driven capacitance enhancement of biomimetic structural SCs. With the synergistic effect of the nature‐inspired spiral grass‐structured design and lamellar grana‐like GQDs/MXene nanohybrids on the trapping and absorption of sunlight, the novel structural photothermal SC exhibits large electrochemical capacitance (10.47 F cm^−2^), outstanding light absorption and light‐to‐heat performance (up to 67.6 °C), a record‐high 3.04‐fold increase in pseudo‐capacitance under one‐sun illumination, record‐high power (223.58 mW cm^−2^) and energy (1.18 mWh cm^−2^) densities, and excellent cycling‐stability (1×10^4^ cycles). Overall, this work paves the way for further improvement of solar thermal energy application and advances novel architecture designs for energy storage and photo‐response devices.

## 2 Results and Discussion

**Figure 1:**
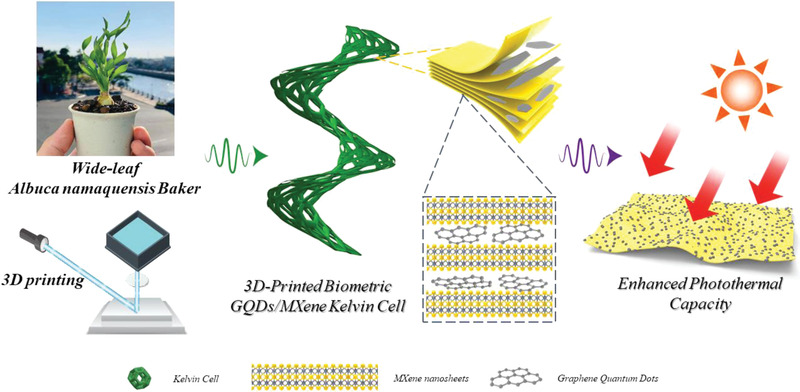
Fabrication schematic illustration of the 3D‐printed biomimetic GQDs/MXene Kelvin cell lattice electrodes.

The design ideals and 3D models of the Wide‐leaf *Albuca namaquensis* Baker‐like biomimetic structures are shown in Figures S1 and S2, Supporting Information. The fabrication process of the 3D‐printed biomimetic GQDs/MXene Kelvin cell lattice electrodes is schematically depicted in **Figure** **1**, and the detailed information is given in the Experimental Section. Briefly, MXene was obtained through etching Ti_3_AlC_2_ in concentrated hydrofluoric acid followed by delamination through sonication. Then, GQDs/MXene nanohybrids ink was prepared by one‐step hydrothermal method. By coating 3D‐printed biomimetic metallic SiOC ceramic torsional Kelvin cell lattices (Figure S3, Supporting Information) with GQDs/MXene nanohybrids, highly photo‐thermal and electroactive Wide‐leaf *Albuca namaquensis* Baker‐like GQDs/MXene electrodes were produced.

**Figure 2:**
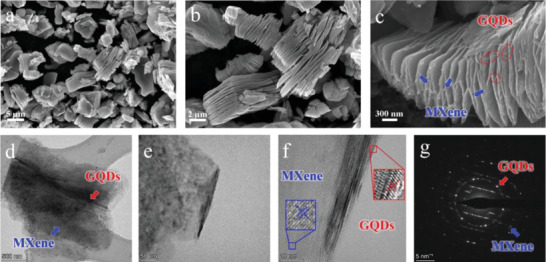
a–c) SEM images of GQDs/MXene nanohybrids. d–f) TEM and HRTEM images of GQDs/MXene nanohybrids. g) SAED pattern.

The morphology and microstructure of the GQDs/MXene nanohybrids were observed by scanning electron microscopy (SEM) and transmission electron microscopy (TEM). As shown in **Figure** **2**a,b, after the introduction of GQDs, the GQDs/MXene nanohybrids maintain the same typical accordion‐like layered structure of pure MXene without any visible change. The high‐magnification SEM image in Figure 2c clearly illustrates that the GQDs and MXene nanosheets are uniformly cross linked, demonstrating the successful engineering of 3D well‐interconnected network, which is advantageous to the ion transport and supercapacitance performance. TEM observations reveal that the as‐synthesized GQDs/MXene displays the ultrathin and transparent feature with the lateral size of ≈2 µm and thickness of ≈3 nm, and both intercrossed flat MXene nanosheets and crumpled GQDs coexist in the GQDs/MXene composite (Figure 2d,e). The clear lattice fringes with d‐spacings of 0.224 and 0.167 nm in the high resolution TEM (HRTEM) image (Figure 2f) and well‐defined diffraction rings in the corresponding selected area electron diffraction (SAED) pattern (Figure 2g) match well with the (008) crystal plane of hexagonal Ti_3_C_2_T*_x_* (JCPDS file No. 52–0875) and the (004) crystal plane of graphene (JCPDS file No. 25–0284), respectively. Additionally, the uniform distribution of Ti, C, O, and F elements in GQDs/MXene is clearly visualized by elemental mapping analysis as presented in Figure S4, Supporting Information. It's worth noting that the existence of O and F elements should be ascribed to the introduced fluorine/oxygen‐containing functional groups such as —OH, =O and —F, which may result in the additional pseudocapacitive behavior. Different from the dense face‐to‐face restacking, the heteroassembly of MXene and GQDs can provide abundant electroactive sites for fast reversible faradic reaction and simultaneously improve the electron/ion transportation for ideal capacitive behavior.

**Figure 3:**
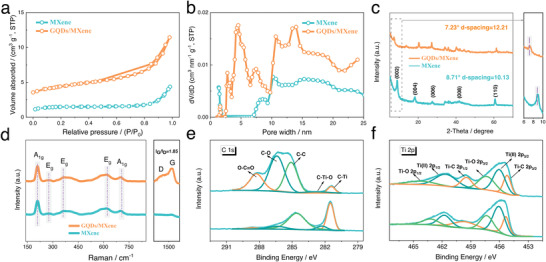
a) N_2_ adsorption–desorption isotherms, b) pore size distribution, c) XRD patterns and d) Raman spectra of MXene and GQDs/MXene; high‐resolution e) C 1s and f) Ti 2p XPS spectra of MXene and GQDs/MXene.

To investigate textural properties of MXene and GQDs/MXene, the nitrogen adsorption and desorption were performed. The N_2_ adsorption/desorption isotherms and corresponding pore size analysis for GQDs/MXene in reference to MXene are shown in **Figures** **3**a and 3b, respectively. As depicted in Figure 3a, different from MXene, the nitrogen sorption of GQDs/MXene shows a typical type‐IV isotherm with H_2_ hysteresis loop in the relative partial pressure region from 0.4 to 0.95, indicating that the porosity mainly consisted of micropores and mesopores. The Brunauer‐Emmett‐Teller (BET) surface area of GQDs/MXene is found to be 32.2 m^2^ g^−1^, about sixfold higher than that of pure Ti_3_C_2_T*_x_* MXene (5.5 m^2^ g^−1^), evidencing the improved exposed surface area of MXene nanosheets. The mesopore/micropore volume ratio of MXene‐GQDs is as high as 2.10, much higher than MXene (1.47). Compared to the porosity distribution of MXene, the GQDs/MXene exhibited abundant large mesopores and a wide pore size distribution from 2 to 25 nm, further suggesting that efficient modification by GQDs as functional spacers facilitate the generation of large layer spacings and diverse pores (Figure 3b). This synergic integration of GQDs into MXene results in more developed hierarchical porosity, allowing for easy electrolytic accessibility of MXene surface sites and enhancing the structural stability during the electrochemical charge‐storage process, thus ensuring superior specific capacitance and rate performance.

The phase composition and crystalline structure of MXene and GQDs/MXene were characterized by X‐ray diffraction (XRD) and Raman measurements. Figure 3c displays the typical XRD patterns of the MXene and GQDs/MXene and enlarged view of the (002) diffraction peaks. As shown in Figure S5, Supporting Information, all the diffraction peaks of the Ti_3_AlC_2_ can be indexed to the pure phase of Ti_3_AlC_2_ (JCPDS file No. 52–0875). No obvious impurity peaks were found. After HF etching, the (104) characteristic diffraction peak of Ti_3_AlC_2_ nearly disappear and the (002) diffraction peak shifts from 9.52° to 8.71°, confirming the successful removal of Al layers and good exfoliation of MXene flakes. A further clear down‐shift to 7.23° is evidenced in the case of GQDs/MXene, indicative of the expansion of the interlayer spacing from 1.01 to 1.22 nm after GQDs introduction, which coincides well with the above TEM observation. The enhanced interlayer spacing of GQDs/MXene results from the synergistic effect of self‐assembly of MXene with GQDs and the intercalation of GQDs between MXene layers, which will guarantee easy ion accessibility and high capacitive performance. The absence of peaks for transition metal oxides in the patterns of GQDs/MXene demonstrates a good retention of the MXene flakes after hydrothermal treatment with no obvious oxidation.^[^ ^17^ ^]^

Raman spectra of MXene and GQDs/MXene are shown in Figure 3d. The two strong vibration modes near 207 and 702 cm^−1^ are A_1g_ symmetry out‐of‐plane vibrations of Ti and C atoms, while the three modes observed at around 282, 374 and 618 cm^−1^ relate to the E_g_ group vibrations, including the in‐plane modes of Ti, C and atoms from surface functional groups, respectively. Besides these peaks, two additional broad bands appear at 1351 and 1573 cm^−1^ for the GQDs/MXene, which could be assigned to the characteristics of D and G bands for graphitic carbon, verifying the coexistence of GQDs with MXene (*I*_G_/*I*_D_ = 1.85). Additionally, the Raman spectrum of GQDs/MXene exhibits no evidence of TiO_2_ (≈150 cm^−1^), demonstrating the good thermal stability and oxidation resistance.^[^ ^9a^ ^]^

X‐ray photoelectron spectroscopy (XPS) was employed to examine the chemical composition and surface electronic states of MXene and GQDs/MXene. The XPS survey spectra (Figure S6a, Supporting Information) indicate that the MXene and GQDs/MXene are mainly composed of Ti, C, O, and F elements. The high‐resolution C 1s core level (Figure 3e) can be deconvoluted into five components located at 281.5, 282.3, 284.8, 286.4 and 288.3 eV, respond to the C—Ti, C—Ti—O, C—C, C—O and O—C=O bonds, respectively. Notably, the atomic percentages of C—O, O—C=O in all fitted components increase from 8.4% and 3.0% for MXene to 42.7% and 20.9% for GQDs/MXene, further demonstrating the successful introduction of GQDs and the interaction between MXene and GQDs. In Figure 3f, the high‐resolution Ti 2p spectra of MXene and GQDs/MXene consist of three doublets centered at 455.2/459.3, 456.1/461.7 and 457.4/463.2 eV corresponding to the Ti—C, Ti(II) and Ti—O signals, respectively.^[^ ^18^ ^]^ For O 1s spectrum of GQDs/MXene, there is no additional oxidation peak at 531 eV, confirming that no obvious surface oxidation has occurred (Figure S6b, Supporting Information).^[^ ^9a^ ^]^

To study the electrochemical performance of GQDs, MXene and GQDs/MXene nanohybrids, 5 mL of pure GQDs ink, pure MXene ink and GQDs/MXene nanohybrids ink with the same concentration of 6 mg mL^−1^ were drop‐casted on the Ag nanowires (AgNWs)‐coated 3D‐printed SiOC plate under the same experimental conditions (detailed in the Experimental Section). The electrochemical measurements of GQDs‐plate, MXene‐plate and GQDs/MXene‐plate electrodes were performed via a three‐electrode system using Pt foil and Ag/AgCl as the counter electrode and reference electrode, respectively, in 1 m Na_2_SO_4_ aqueous solution. The GQDs‐plate electrode displays nearly rectangular cyclic voltammogram (CV) curves at 5 mV s^−1^ and triangular galvanostatic charge–discharge (GCD) profiles at 0.1 A cm^−2^ (Figure S7a,b, Supporting Information), indicating typical electrical double‐layer capacitor behavior, while the obvious redox peaks in the CV curves and nonlinearity in the GCD curves of both MXene‐plate and GQDs/MXene‐plate electrodes verify the pseudocapacitance behavior. Moreover, significant increases in the integrated CV area and charging/discharging time for the GQDs/MXene‐plate electrode can be observed, which means much higher specific capacitance for GQDs/MXene. As expected, the rate capability of GQDs/MXene‐plate electrode is found to be significantly boosted in comparison with MXene‐plate electrode (Figure S7c, Supporting Information). When the current density increases from 5 to 500 mA cm^−2^, the specific areal capacitance (*C*_A_) of GQDs/MXene‐plate electrode decreases from 4.11 to 1.71 F cm^−2^ (a capacitance retention of 41.9%), while the MXene‐plate electrode displays a poor capacitance retention of 29.5%. Meanwhile, as shown in Figure S7d, Supporting Information, the charge transfer resistance of GQDs/MXene‐plate electrode (7.22 Ω) is reduced dramatically compared with MXene‐plate electrode (13.71 Ω). That's because the nanohybrids of 0D GQDs and 2D MXene can convert the planar configuration of MXene into a highly conductive 3D interconnected network with open and porous architectures to overcome the disreputable restacking problem of MXene and obtain improved capacitive/rate performance. In view of the carried measurements, GQDs/MXene nanohybrids, with its superior structural characteristics and electrochemical properties, fits best as a prospective electrode material for the construction of a bionic structural photothermal SC.

**Figure 4:**
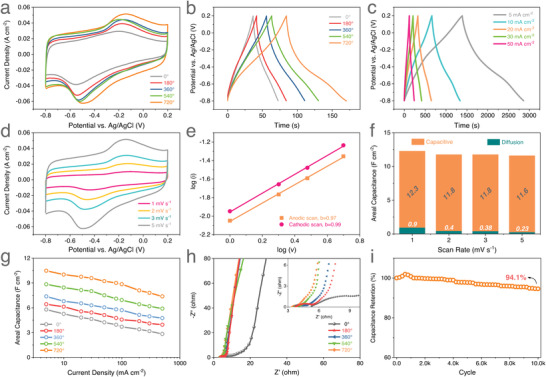
Electrochemical performance of the 3D‐printed biomimetic GQDs/MXene torsional Kelvin cell lattice electrodes with different twist angles. a) CV curves of the biomimetic GQDs/MXene electrodes at 5 mV s^−1^. b) GCD curves of the biomimetic GQDs/MXene electrodes at 0.1 A cm^−2^. c) GCD curves of the GQDs/MXene‐720° electrode at different current densities. d) CV curves of the GQDs/MXene‐720° electrode at different scan rates. e) Logarithmic of the anodic and cathodic peak currents at different scan rates for GQDs/MXene‐720° electrode. f) Capacitance contribution for the GQDs/MXene‐720° electrode at different scan rates. g) *C*_A_ of the biomimetic GQDs/MXene electrodes obtained at different current densities. h) Nyquist plots of the biomimetic GQDs/MXene electrodes. i) Cyclic stability of the GQDs/MXene‐720° electrode at 500 mA cm^−2^ for 10 000 cycles.

To further explore the effect of bionic structure design on the electrochemical performance of structural electrodes, 5 mL GQDs/MXene nanohybrids ink with a concentration of 6 mg mL^−1^ were drop‐casted on the AgNWs‐coated 3D‐printed biomimetic SiOC torsional Kelvin cell lattices with different twist angles, respectively, under the same experimental conditions (detailed in the Experimental Section). As exhibited in Figure S8, Supporting Information, the GQDs/MXene nanohybrids is evenly distributed and firmly attached to the 3D‐printed bionic Kelvin cell lattice skeleton, ensuring good electrical conductivity, high stability, and promising capacity. The electrochemical performances of the 3D‐printed biomimetic GQDs/MXene electrodes with different twist angles (0°, 180°, 360°, 540°, 720°) were measured using a three‐electrode system with 1 m Na_2_SO_4_ aqueous solution as electrolyte. The CV curves of 3D‐printed biomimetic GQDs/MXene electrodes with different twist angles collected at a fixed scan rate of 5 mV s^−1^ within the voltage ranging from −0.8 to 0.2 V versus Ag/AgCl reference are given in **Figure** **4**a. The presence of a pair of distinct broad redox peaks at −0.6 to −0.1 is evident in all CV curves, which originates from the protonation and change in the oxidation status of Ti atoms, suggesting the pseudocapacitive charge storage mechanism. Figure 4b compares the GCD curves of the 3D‐printed biomimetic GQDs/MXene electrodes with different twist angles measured at a constant current density of 100 mA cm^−2^. All GCD curves are highly symmetric with a slight distortion from the ideal triangle shape in all cases and the corresponding voltage plateaus are almost consistent with the CV curves, further demonstrating the pseudocapacitive nature of the 3D‐printed biomimetic GQDs/MXene electrodes. Moreover, the integrated CV area for GQDs/MXene‐720° electrode is much larger than that of other electrodes, while the charge/discharge curves for GQDs/MXene‐720° electrode also exhibit a much longer charging/discharging time and significantly lower voltage drop, revealing its highest specific capacitance and best electrochemical properties. This strongly confirms the effectiveness of the structural engineering from 2D periodic structure into 3D torsional architecture in facilitating the ion transport and improving the ion accessibility.

Figure 4c illustrates the GCD profiles of the GQDs/MXene‐720° electrode at different current densities from 5 to 50 mA cm^−2^. The GCD curves are highly symmetric at various current densities investigated with negligible IR drop, illustrating great reversibility, and pseudocapacitive properties. Meanwhile, with an increase of scan rate to 5 mV s^−1^, the CV curves of the GQDs/MXene‐720° electrode can still maintain similar shapes with an inappreciable shift of the anodic and cathodic peaks, indicating its highly capacitive nature with fast ion response and satisfactory rate capability (Figure 4d). Generally, the CV data were analyzed according to the power–law relationship (*i* = *aυ^b^*) to determine the charge storage mechanism of the electrodes. Both *a* and *b* are adjustable values. The *b*‐value can be obtained from the slope of the log(*i*) versus log(*υ*) plot. For a diffusion‐controlled intercalation/deintercalation process, the *b*‐value is 0.5, while for an ideal surface‐controlled capacitive (electrical double‐layer capacitor and surface pseudocapacitance) process, the *b*‐value is 1.0. The logarithmic of the anodic and cathodic peak currents from 1 to 5 mV s^−1^ for GQDs/MXene‐720° electrode are plotted in Figure 4e. The *b*‐values of 0.97 and 0.99 are obtained for the anodic and cathodic peaks, respectively, which implies that the kinetics of GQDs/MXene‐720° are mainly non‐diffusion limited, thus providing superior rate capability. Figure S9, Supporting Information, provides the potential profile for the current from capacitive effects (masked area) of the GQDs/MXene‐720° electrode at different scan rates (1, 2, 3, and 5 mV s^−1^). Figure 4f segregates the capacitive and diffusion‐controlled contributions to the total capacity at different scan rates. The contribution ratio of the capacitive process gradually improves from 93% to 99% with increased scan rates, revealing the dominant capacitive charge–storage mechanism, which would lead to the superior electrochemical performance of rate capability and long‐term cycling stability. These results further demonstrate that the high porosity of 3D torsional architecture can significantly enhance the diffusion and transportation of electrolyte ions, and increase the surface area accessible to the electrolyte ions during the energy storage process, and thus boost the total capacitance and rate performance.

The variations of *C*_A_ against current density in the range from 5 to 500 mA cm^−3^ for the printed biomimetic GQDs/MXene electrodes are calculated and plotted in Figure 4g. Notably, an outstanding *C*_A_ of 10.47 F cm^−2^ for GQDs/MXene‐720° electrode is calculated at 5 mA cm^−2^ which is approximately twofold increase compared with 5.68 F cm^−2^ for GQDs/MXene‐0°, and superior to 6.42, 7.23 and 8.48 F cm^−2^ for GQDs/MXene‐180°, GQDs/MXene‐360° and GQDs/MXene‐540°, respectively. The *C*_A_ of the GQDs/MXene‐720° electrode at the current densities of 10, 20, 30, 50, 80, 100, 200, 300 and 500 mA cm^−2^ are found to be 10.02, 9.75, 9.59, 9.21, 9.01, 8.85, 8.10, 7.80 and 7.37 F cm^−2^, respectively. Although the value of *C*_A_ decreases with the increasing of current density, the GQDs/MXene‐720° electrode can still deliver a high *C*_A_ of 7.37 F cm^−2^ with a capacitance retention of 70.4% even when the current density increases to an ultrahigh value of 500 mA cm^−2^, suggestive of rapid charge transfer behavior and excellent rate capability due to its 3D open torsional architecture. In contrast, GQDs/MXene‐0° and GQDs/MXene‐180° electrodes demonstrate poor rate capabilities of 49.1% and 61.5% capacitance retention, respectively.

To further illuminate the kinetics of charge transfer and ion transport of the 3D‐printed biomimetic torsional Kelvin cell lattice electrodes, the electrochemical impedance spectroscopy (EIS) measurements were performed. As shown in Figure 4h, all of the Nyquist plots feature a semi‐circle in the high‐frequency region, followed by an inclined line in the low‐frequency region. It is clear that in comparison to other torsional Kelvin cell lattices, GQDs/MXene‐720° delivers the smallest equivalent series resistance (intercept of *Z* real axis) of 1 Ω and significantly diminished interfacial charge‐transfer resistance (diameter of semicircle) of 0.5 Ω, indicative of the enhanced ion‐transfer kinetics. Furthermore, the slope of low‐frequency tail of GQDs/MXene‐720° is evidently greater than that of other electrodes, implying the faster ionic diffusion process and better supercapacitive behavior. The improved ion transport behavior is mainly attributed to the elaborate 3D electrode structure design that provides dramatically increased exposed surface area sites and shortened ion transport pathways.

The cycling performance of the GQDs/MXene‐720° electrode was evaluated by GCD measurements at 0.5 A cm^−2^ and the results are shown in Figure 4i. Impressively, after 10 000 continuous cycles, a favorable capacitance retention of 94.1% is achieved while the coulombic efficiency remains constant, justifying the remarkable durability and electrochemical reversibility of the devised GQDs/MXene‐720° electrode.

**Figure 5:**
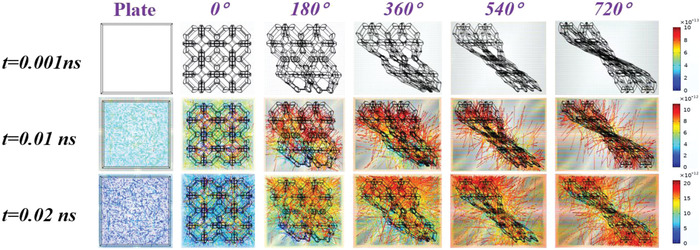
Simulation results of the light transport within the biomimetic torsional Kelvin cell lattice electrodes with different twist angles.

To investigate the light‐absorption and light‐trapping properties of the Wide‐leaf *Albuca namaquensis* Baker‐like structures, we simulated the light transport within the bionic torsional Kelvin cell lattice electrodes with different twist angles. As shown in **Figure** **5**, when *t* = 0.01 ns, the light reflection inside the bionic structures is significantly enhanced compared with the plate structure. Meanwhile, with the increase of the twist angle, the light trapping effect is continuously improved, and the light distribution gradually trends to be uniform, which are conducive to the uniform increase of the electrode temperature by the photothermal effect. With the increasing time, the light trapping and uniform reflection effects of the biomimetic structures with high twist angle become more significant, which can prove that the well‐designed biomimetic structural electrode is promising in the application of 3D photothermal energy storage devices.

**Figure 6:**
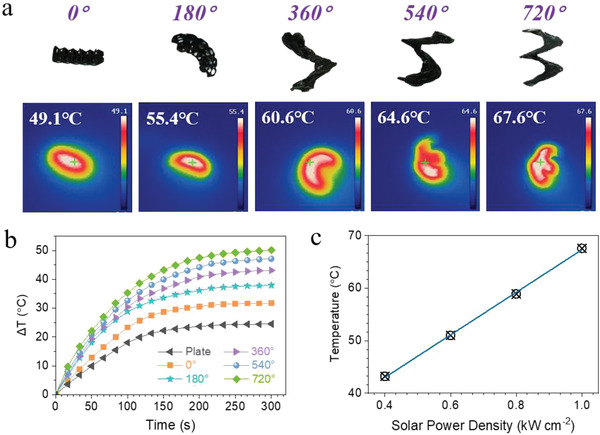
a) Infrared images of the bionic structural electrodes with different twist angles under 1 solar illumination. b) Δ*T*‐time plots of the bionic structural electrodes with different twist angles under 1 solar illumination. c) Surface temperature of the GQDs/MXene‐720° electrode under solar illumination at different power densities.

To evaluate the photothermal performance of the 3D‐printed biomimetic GQDs/MXene torsional Kelvin cell lattice electrodes, the bionic structural electrodes with different twist angles were exposed to solar light irradiation, and their surface temperatures as a function of time were recorded by the infrared camera. When exposed to 1 sun illumination, the infrared images clearly visualize the increase of electrode temperature upon the irradiation (**Figure** **6**a), verifying the photothermal effect of GQDs/MXene electrodes. Under 1‐sun illumination for 5 min, the surface temperatures are 41.9, 49.1, 55.4, 60.6, 64.6 and 67.6 °C for the GQDs/MXene‐plate, 0°, 180°, 360°, 540° and 720° electrodes, respectively. Interestingly, as illustrated in Figure 6b, the surface temperature changes (Δ*T*) of the bioinspired structures are consistently higher than that of the plate structure and an obvious enhancement in photothermal conversion capability could be observed as the twist angle increases. Furthermore, as depicted in Figure 6c, the surface temperature of the GQDs/MXene‐720° electrode increases from 43.2 to 67.6 °C, when the power density of the solar light increases from 0.4 to 1 kW m^−2^. The superior photothermal capability of GQDs/MXene‐720° electrode should be attributed to the synergistic effect of high photothermal conversion efficiency of the lamellar grana‐like GQDs/MXene nanohybrids and light‐trapping biomimetic structures that allow incident light to undergo multiple internal reflections for enhanced light absorption.

**Figure 7:**
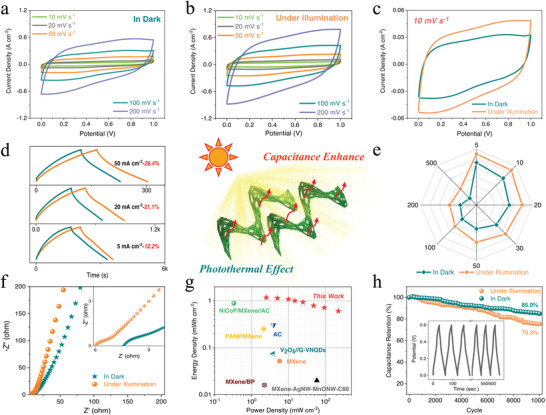
Electrochemical performance of the biomimetic structural GQDs/MXene photothermal SCs. CV profiles of the GQDs/MXene‐720° SC at different scan rates a) in the dark and b) under 1 solar illumination. c) Comparative CV curves at 10 mV s^−1^ of the device in the dark and under 1 solar illumination. d) GCD curves of the SC at various current densities in the dark and under 1 solar illumination. e) Rate capability and f) Nyquist plots of the device in the dark and under 1 solar illumination. g) Ragone plot of the biomimetic structural GQDs/MXene photothermal SC in comparison with other recently reported 3D‐printed or MXene‐based SCs. h) Cycling performance of the device at 0.2 A cm^−2^ in the dark and under 1 solar illumination.

Photothermal Ss were constructed by assembling two identical 3D‐printed biomimetic structural GQDs/MXene‐720° electrodes with 1 m Na_2_SO_4_ electrolyte (as shown in Figure S10, Supporting Information). **Figures** **7**a and 7b display the CV characteristics of the as‐assembled GQDs/MXene‐720° SC with a 1.0 V potential window at various scan rates from 10 to 200 mV s^−1^ in the dark and under 1 solar illumination, respectively. To better understand the effect of solar irradiation on the performance of bionic structural photothermal SC, Figure 7c compares the CV profiles at a scan rate of 10 mV s^−1^ in the dark and under solar light illumination. Evidently, regardless of the scan rate, the CV curves under solar light illumination exhibit similar typical shapes of pseudocapacitive behavior (a pair of redox peaks) to those in the dark but with higher current responses and larger integrated areas, thereby indicating no new types of charge storage reactions but significantly increased capacitance and excellent reversibility under solar illumination due to the photothermal effect. Furthermore, no noticeable peak shape distortion is found as the scan rate increases, demonstrating the great endurance of a high rate.

The GCD profiles of the obtained photo‐responsive device conducted at different current densities (5, 20, 50 mA cm^−2^) in the dark and under solar light illumination are shown in Figure 7d, confirming the same pseudocapacitive behavior as presented in CV measurements. Most notably, the GCD curves of the symmetric GQDs/MXene‐720° SC under solar light illumination maintain well their original nearly symmetrical subtriangular shapes in the dark, but with evidently increased charge–discharge time and decreased ohm‐drop, revealing a unique photo‐responsive feature generated from both high‐efficiency light‐trapping biomimetic structure and strong photothermal effect of GQDs/MXene nanohybrids. The enhancement of the capacitance gradually improves from 13.8% (from 7.48 to 8.51 F cm^−2^) to 26.4% (from 5.57 to 7.04 F cm^−2^) as the current density increases from 5 to 50 mA cm^−2^, suggesting that the solar thermal‐driven capacitance enhancement is more significant at a high rate.

The variation of *C*_A_ value with applied current densities (from 5 to 500 mA cm^−2^) for the biomimetic structural GQDs/MXene photothermal SCs in the dark and under 1 solar illumination are plotted in Figure 7e. Obviously, the *C*_A_ value decreases with increasing current density owing to the kinetic limitations.^[^ ^19^ ^]^ However, the symmetric GQDs/MXene‐720° SC delivers an outstanding rate capability of 53.0% at 500 mA cm^−2^ with a high *C*_A_ of 4.02 F cm^−2^ in the dark, much higher than that of plate structure and periodic Kelvin cell lattice SCs (details in Figure S11, Supporting Information). More importantly, as the current density increases to 500 mA cm^−2^, the capacitance retention of the biomimetic structural SC could even reach 64.0% (5.45 F cm^−2^) under 1‐sun illumination, which is significantly superior to that of a plate structure (1.52 F cm^−2^, 43.8%) and periodic Kelvin cell lattice (2.08 F cm^−2^, 44.5%) SCs, highlighting the crucial role of the high‐efficiency light‐trapping Wide‐leaf *Albuca namaquensis* Baker‐like biomimetic structure (Figure S12, Supporting Information).

Moreover, Figures S13–S15, Supporting Information, depict the *C*_A_ values of the biomimetic structural SCs with different twist angles at 5, 50, and 500 mA cm^−2^ in the dark and under solar light illumination, respectively. It can be clearly observed that compared with the plate structure SC in the dark, the biomimetic torsional Kelvin cell lattice GQDs/MXene‐720° SC possesses ≈161.0% enhancement of *C*_A_ (from 3.26 to 8.51 F cm^−2^) at 5 mA cm^−2^, 2.17 times enhancement of *C*_A_ (from 2.22 to 7.04 F cm^−2^) at 50 mA cm^−2^ and 3.04 folds enhancement of *C*_A_ (from 1.35 to 5.45 F cm^−2^) at 500 mA cm^−2^ under solar light illumination, justifying the significant solar thermal‐driven capacitance enhancement of biomimetic structural SCs, especially at a high rate. The above results demonstrate the remarkable structure‐enhanced photothermal response and outstanding electrochemical performance of the biomimetic structural GQDs/MXene photothermal SCs, enabling a high energy density.

To provide further insights, EIS spectra of the bionic structural photothermal SCs in the dark and under solar light illumination were investigated to quantify the resistance at the electrode/electrolyte interface (Figure 7f). As shown, the symmetric GQDs/MXene‐720° SC displays a slightly higher slope at low frequencies and depressed semicircle with a smaller radius in the high frequency region under 1‐sun illumination than that in the dark, indicating more capacitive behavior and facile accessibility of the electrolyte's ions to charge storage sites under solar illumination. Moreover, the equivalent series resistance and charge transfer resistance of the photothermal SC are ≈6.64 and ≈1.65 ohms in the dark, which decrease to 6.05 and 0.87 ohms under solar illumination, respectively. Thus, the mechanism of the capacitance enhancement of the 3D‐printed biomimetic structural GQDs/MXene photothermal SCs under solar light illumination can be ascribed to the synergistic effect of a high light‐trapping property of biomimetic structures and strong photothermal conversion of lamellar grana‐like GQDs/MXene nanohybrids. The photo‐responsive properties of GQDs/MXene nanohybrids result in high conductivity and fast ion transport behavior under solar light illumination, while the unique architecture of the Wide‐leaf *Albuca namaquensis* Baker‐like biomimetic structure not only provides porous networks for sufficient electrolyte/electrode contact, but also allows incident light to undergo multiple internal reflections for the enhanced photothermal effect of GQDs/MXene nanohybrids. These characteristics in 3D‐printed biomimetic GQDs/MXene torsional Kelvin cell lattice photothermal SCs work to improve both energy density and power density.

Areal energy density and power density are crucial factors to evaluate the performance of electrochemical energy storage devices. Figure 7g depicts the energy–power performance metrics of the 3D‐printed biomimetic structural GQDs/MXene photothermal SCs in the form of a Ragone plot. It showed that the bionic structural photothermal SC possesses a high areal energy density of 1.18 mWh cm^−2^ at a corresponding power density of 2.49 mW cm^−2^ and retains an energy density of 0.61 mWh cm^−2^ at a maximum power density of 223.58 mW cm^−2^. These values are vastly superior to other recently reported 3D‐printed or MXene‐based SCs, such as 3D‐printed NiCoP/MXene//AC asymmetrical SC (0.89 mWh cm^−2^ at 0.33 mW cm^−2^),^[^ ^20^ ^]^ 3D‐printed MXene‐N based SC (0.42 mWh cm^−2^),^[^ ^21^ ^]^ fused deposition modeled AC SC (306 µWh cm^−2^ at 3.9 mW cm^−2^),^[^ ^22^ ^]^ MNCFT//ONCFT asymmetrical SC (277.3 µWh cm^−2^),^[^ ^23^ ^]^ PANI//MXene asymmetrical SC (252 µWh cm^−2^ at 2.12 mW cm^−2^),^[^ ^24^ ^]^ 3D‐printed V_2_O_5_//G‐VNQDs micro asymmetrical SC (73.9 µWh cm^−2^ at 3.77 mW cm^−2^),^[^ ^25^ ^]^ direct‐ink‐written MXene‐based micro SC (51.7 µWh cm^−2^ and 5.7 mW cm^−2^),^[^ ^26^ ^]^ 3D‐printed MXene‐AgNW‐MnONW‐C60 micro SC (19.2 µWh cm^−2^ at 58.3 mW cm^−2^),^[^ ^27^ ^]^ direct‐laser‐writing MXene/BP micro SC (15.94 µWh cm^−2^ at 2.22 mW cm^−2^),^[^ ^28^ ^]^ stereolithographic printed rGO SC (8 µWh cm^−2^ at 0.75 mW cm^−2^),^[^ ^29^ ^]^ screen‐printed MnHCF‐MnO*_x_*/ErGO SC (2.3 µWh cm^−2^ at 0.5 mW cm^−2^),^[^ ^30^ ^]^ extrusion‐printed all‐MXene micro SC (0.32 µWh cm^−2^ at 11.4 µW cm^−2^)^[^ ^31^ ^]^ and inkjet‐printed MXene SC (0.0043 µWh cm^−2^ at 0.077 µW cm^−2^).^[^ ^32^ ^]^

The cycling stability of the biomimetic structural GQDs/MXene photothermal SC was assessed at a high current density of 0.2 A cm^−2^, as exhibited in Figure 7h. When cycled at 200 mA cm^−2^ in the dark for 10 000 cycles, the symmetric GQDs/MXene‐720° SC maintains a high capacity of 4.03 F cm^−2^, delivering an excellent capacity retention of 85.0%. In contrast, the bionic structural photothermal SC shows a large capacity decay under 1 solar illumination, with a capacity retention of ≈75.3% after 10 000 cycles (from 5.88 to 4.43 F cm^−2^), which is due to the increased reaction rates of side reactions at increased temperature under solar light illumination.^[^ ^5a^ ^]^

**Figure 8:**
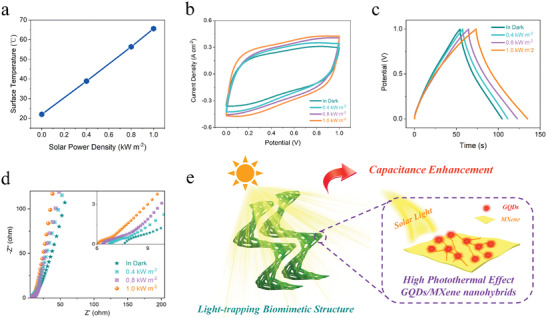
Electrochemical performance of the biomimetic structural GQDs/MXene‐720° photothermal SC under sunlight with different power densities. a) Surface temperature, b) CV curves at 100 mV s^−1^, c) GCD curves at 100 mA cm^−2^, and d) Nyquist plots of the symmetric GQDs/MXene‐720° SC under solar illumination at different power densities. e) Schematic illustration of the structure‐enhanced solar thermal‐driven capacitance enhancement of the biomimetic structural GQDs/MXene photothermal SC.

The electrochemical performance of the biomimetic structural GQDs/MXene‐720° photothermal SC under sunlight with different power densities was also tested. As displayed in **Figure** **8**a, the surface temperature of the biomimetic structural SC gradually increases with increasing solar power density and reaches 66.1 °C under 1‐sun illumination. In Figure 8b, the current response and enclosed area of the CV curves (100 mV s^−1^) progressively increase with the increase of solar power density, while maintaining a similar shape of the CV curves, demonstrating the same reaction type and an increasing capacitance with the rising power density. Figure 8c compares the GCD curves of the biomimetic structural GQDs/MXene‐720° photothermal SC at 100 mA cm^−2^ under solar illumination at different power densities. All GCD curves present a nearly isosceles triangle shape, proving the high redox reversibility and good coulombic efficiency. Moreover, in agreement with CV results, the discharge time of the biomimetic structural SC increases gradually with the increase of power density, indicating an increasing capacitance with increasing solar power density. From the Nyquist plots in Figure 8d, it can be seen that both equivalent series resistance and charge transfer resistance decrease continuously with rising solar power density.

The mechanism for the solar thermal‐driven capacitance enhancement of the 3D structural GQDs/MXene  can be explained by the biomimetic structure‐enhanced photothermal effect, as shown in Figure 8e. First, the unique light‐absorbing and light‐trapping properties of the Wide‐leaf *Albuca namaquensis* Baker‐like biomimetic structure (macroscale “leaf blade”) allow incident light to undergo multiple internal reflections. As a result, under the same light irradiation, more solar irradiation can be converted into heat by photothermal materials, thus inducing higher light‐to‐heat conversion efficiency. Second, the grana‐like GQDs/MXene nanohybrids (microscale “chloroplast”) convert the planar configuration of MXene into a 3D interconnected structure with open architectures and wide absorption range to overcome the disreputable restacking problem and obtain improved photothermal and capacitive performances. Third, the homogeneous distribution of GQDs on the surface of MXene is beneficial for uniform heating and rapid charge transfer.

## 3 Conclusion

In summary, we designed a simple and generalized method to construct GQDs/MXene hybrid nanocoating (“chloroplast”) on 3D‐printed torsional Kelvin cell lattice arrays (“leaf blade”) to achieve biomimetic photothermal electrodes, imitating the hierarchical structure of *Albuca namaquensis* Baker's spiral leaves from macro to micro scale. This novel nature‐inspired structural GQDs/MXene photothermal electrode can implement the strong coupling of large‐capacitance high‐photothermal conversion efficiency GQDs/MXene nanohybrids as well as effective light‐trapping and light‐absorption biomimetic torsional Kelvin cell lattice arrays. The biomimetic structural photothermal SC unveils exceptional capacitive performance (10.5 F cm^−2^), high equilibrium temperature (67.6 °C) and record‐high 3.04 times capacitance enhancement under solar light illumination, and a maximum energy density of 1.18 mWh cm^−2^ and a power density of 223.58 mW cm^−2^. Considering these remarkable achievements, we anticipate that the MXene‐based photothermal SC and its biomimetic structure design will advance new‐generation multifunctional electrode architecture and guide the substantial progress in the fields of solar thermal systems, energy storage devices, electromagnetic shielding appliances, light sensors, electrochemical actuators and biomedical robotics.

## 4 Experimental Section

### 3D Printing of Biomimetic SiOC Torsional Kelvin Cell Lattices

The Kelvin cell with √2/2 mm strut edge length and 400 µm strut diameter was used as the basic unit cell of the lattice structure in this study. Five kinds of 3D torsional Kelvin cell lattice structures with different twist angles of 0°, 180°, 360°, 540°, and 720° were designed and studied. The 3D design was done in SolidWorks and later processed in dlpui (slicing software) to create a .sky file for printing. The photocurable preceramic polymer was prepared as in a previous work. The polymer‐derived SiOC torsional Kelvin cell lattices were printed by a digital light processing‑based 3D printer (Longer3D, CeraForm100), and then pyrolyzed at 900 °C for 4 h in N_2_ atmosphere with a heating rate of 1 °C min^−1^.

### Preparation of AgNWs‐Coated 3D‐Printed Biomimetic SiOC Torsional Kelvin Cell Lattices

The AgNWs‐coated 3D‐printed biomimetic SiOC torsional Kelvin cell lattices were fabricated by a simple facile one‐pot synthesis strategy. First, 0.36 g of PVP and 0.24 g of AgNO_3_ were dissolved in 60 mL of ethylene glycol under gentle stirring. After that, 0.4 mg of FeCl_3_ was added into the above solution suspension and stirred for another 20 min. Next, the printed SiOC torsional Kelvin cell lattices were immersed in the prepared solution and transferred together into a 100 mL Teflon‐lined stainless autoclave. The hydrothermal reaction was conducted at 130 °C for 12 h. The AgNWs‐coated 3D‐printed biomimetic SiOC torsional Kelvin cell lattices were obtained after being washing by deionized water and ethanol for several times.

### Preparation of GQDs/MXene Nanohybrids

To synthesize the multilayer MXene feedstock, 1 g fresh Ti_3_AlC_2_ was etched by a mixture of 1 g LiF and 20 mL 9 m HCL solution in a polytetrafluoroethylene bottle and then kept at 35 °C with continuous stirring for 24 h. Afterwards, the MXene suspension was centrifuged (3500 rpm) and rinsed with distilled water for several times until the pH was higher than 5.5. Finally, multilayer MXene was obtained by freeze‐drying. The GQDs were prepared by a hydrothermal method using glucose as the raw material. Typically, 0.075 g of glucose was dissolved into 75 mL deionized water with constant stirring to obtain a clear solution. Subsequently, the obtained mixed solution was transferred into a Teflon‐lined stainless steel autoclave (100 mL) and heated at 150 °C for 10 h. For further purification of GQDs, the resulting light yellow solution was dialyzed in a dialysis bag (retained molecular weight: 3500 Da) for 48 h. The purified black GQDs were dissolved in distilled water to prepare GQD solution with a concentration of 0.2 mg mL^−1^. The GQDs/MXene nanohybrids were prepared by a simple hydrothermal process. Typically, 60 mg of multilayer MXene was dispersed into 15 mL of GQDs solution with vigorous stirring for 1 h to form a homogeneous suspension. Next, the suspension was sealed in a 30 mL Teflon‐lined autoclave and maintained at 180 °C for 6 h. After centrifugation, the product was washed with ethanol and deionized water for several times, and dried under vacuum at 60 °C for overnight.

### Drop‐Casting of GQDs/MXene on AgNWs‐Coated 3D‐Printed Biomimetic SiOC Torsional Kelvin Cell Lattices

The GQDs/MXene nanohybrids ink with a concentration of 6 mg mL^−1^ was prepared by dispersing 0.06 g GQDs/MXene powder in a 10 mL mixed solvent of water and alcohol (V/V = 1:1) with ultrasonic treatment for 4 h. 3D‐printed biomimetic SiOC torsional Kelvin cell lattices were directly soaked in the ink for 60 s and then dried in a vacuum oven at 60 °C. The dip–dry process was repeated for several times to guarantee a uniform coating of GQDs/MXene. Each structural electrode prepared for electrochemical experiments used 5 mL of GQDs/MXene nanohybrids ink to ensure the same GQDs/MXene loading. The obtained electrodes with different twist angles were labeled as GQDs/MXene‐*n* (*n* = 0°, 180°, 360°, 540°, 720°). For comparison, 5 mL of pure GQDs ink, pure MXene ink and GQDs/MXene nanohybrids ink with the same concentration of 6 mg mL^−1^ were drop‐casted on the AgNWs‐coated 3D‐printed SiOC plate under the same experimental conditions, denoted as GQDs‐plate, MXene‐plate and GQDs/MXene‐plate, respectively.

### Materials Characterization

SEM images were acquired using a FEI Helio G4 CX microscope operated at 25 kV. TEM images were obtained on a FEI Themis Z TEM system with an accelerating voltage of 300 kV. N_2_ adsorption/desorption isotherms were measured on Quantachrome Autosorb‐1‐MP gas adsorption analyzer at 77 K after being degassed in vacuum at 200 °C for 10 h. The BET surface areas and pore size distributions were calculated by the Brunauer–Emmett–Teller equation and density functional theory method, respectively. A Bruker D8 DISCOVER A25 diffractometer with Cu K*α* radiation (*λ* = 1.5418 Å) was used for collecting XRD patterns. Raman spectra were recorded on a Renishaw 1000 with a 633 nm laser. XPS analysis was performed on a Shimadzu AXIS Supra spectrometer with an Al K*α* source (1486.7 eV). The real‐time temperatures and infrared images were recorded using a FLUKE Ti 10 infrared camera.

### Finite Element Simulation

Light transport in biomimetic torsional Kelvin cell lattice structures was simulated by using Comsol Multiphasics software. Geometrical optics was used to model the transmission of light inside the bionic structures. The rays with specified vacuum wavelength (660 nm) were released into the model in parallel, and the light propagation range was constrained by the geometric boundary of the model. Without considering the thermal effect, the refractive index, time step and out time were 50 000, 0.001 ns and 10 ns, respectively (at 293.15 K). Monte Carlo ray tracing was used to produce global illumined images of 3D objects according to the principle of optics.

### Electrochemical Measurements

All the electrochemical measurements were carried out on the electrochemical workstation (CHI 760 E). Prior to assembling a full cell, GQDs/MXene torsional Kelvin cell lattice electrodes were tested in 1 m Na_2_SO_4_ electrolyte using a conventional three‐electrode system where Pt foil and Ag/AgCl were used as the counter electrode and reference electrode, respectively. Two identical 3D‐printed biomimetic structural GQDs/MXene electrodes, Celgard 2400 separator and 3 mL 1 m Na_2_SO_4_ electrolyte were sealed in a cylindrical PVC cell by applying soft‐package technology to assemble the photothermal SCs. Before the assembly, the electrodes and the separator were immersed in electrolyte for several minutes to achieve a good attachment and penetration. The photo‐responsive properties of biomimetic structural SCs were characterized by recording their electrochemical performance by using electrochemical working station with Ss illuminated under simulated solar light irradiation with an intensity of 1 kW m^−2^ (calibrated by using a silicon reference solar cell) which was provided by a PLS‐SXE300D solar simulator. The EIS was recorded in a frequency range from 100 kHz to 0.01 Hz with a voltage amplitude of 5.0 mV. The long‐term stability was evaluated by chronoamperometry measurement at 0.5 A cm^−2^.

*C*_A_ (F cm^−2^) of electrode as well as device were calculated from the GCD curves using the following equation:

(1) $$C_{A}=I\Delta t/A\Delta V$$

where, Δ*V* is potential/voltage window (V), *A* is basal area of the individual electrode or final symmetric cell (cm^−2^), Δ*t* is discharge time in seconds, and *I* is discharge current in amperes.

The specific areal energy (*E*, mWh cm^−2^) and power density (*P*, mW cm^−2^) of the device were obtained from the following equations:

(2) $$E=C_{A}{\left(\Delta V\right)}^{2}/2$$

(3) P=3600E/Δt

## Conflict of Interest

The authors declare no conflict of interest.

## Supporting information

Supporting Information

## Data Availability

The data that support the findings of this study are available from the corresponding author upon reasonable request.
