# Supplementary material for: Nature‐Inspired 3D Spiral Grass Structured Graphene Quantum Dots/MXene Nanohybrids with Exceptional Photothermal‐Driven Pseudo‐Capacitance Improvement
Source: Adv Sci (Weinh). 2022 Aug 26;9(30):2204086. doi: 10.1002/advs.202204086 (PMC9596846; doi:10.1002/advs.202204086)
Supplement: Supplementary file 1 — Supporting Information [file ADVS-9-2204086-s001.pdf]

ADVANCED SCIENCE
Open Access

Supporting Information

for *Adv. Sci.*, DOI 10.1002/advs.202204086

Nature-Inspired 3D Spiral Grass Structured Graphene Quantum Dots/MXene Nanohybrids with Exceptional Photothermal-Driven Pseudo-Capacitance Improvement

Peng Chang, Hui Mei, Yu Zhao, Longkai Pan, Minggang Zhang, Xiao Wang, Laifei Cheng and Litong Zhang*

Nature-inspired 3D Spiral Grass Structured Graphene Quantum Dots/MXene Nanohybrids with Exceptional Photothermal-driven Pseudo-capacitance Improvement

Peng Chang, Hui Mei, Yu Zhao, Longkai Pan, Minggang Zhang, Xiao Wang, Laifei Cheng, Litong Zhang*

Dr. P. Chang, Prof. H. Mei, Dr. Y. Zhao, Dr. L. Pan, Dr. M. Zhang, X. Wang, Prof. L. Cheng, Prof. L. Zhang

Science and Technology on Thermostructural Composite Materials Laboratory, School of Materials Science and Engineering, Northwestern Polytechnical University, Xi'an 710072, China

E-mail: meihui@nwpu.edu.cn

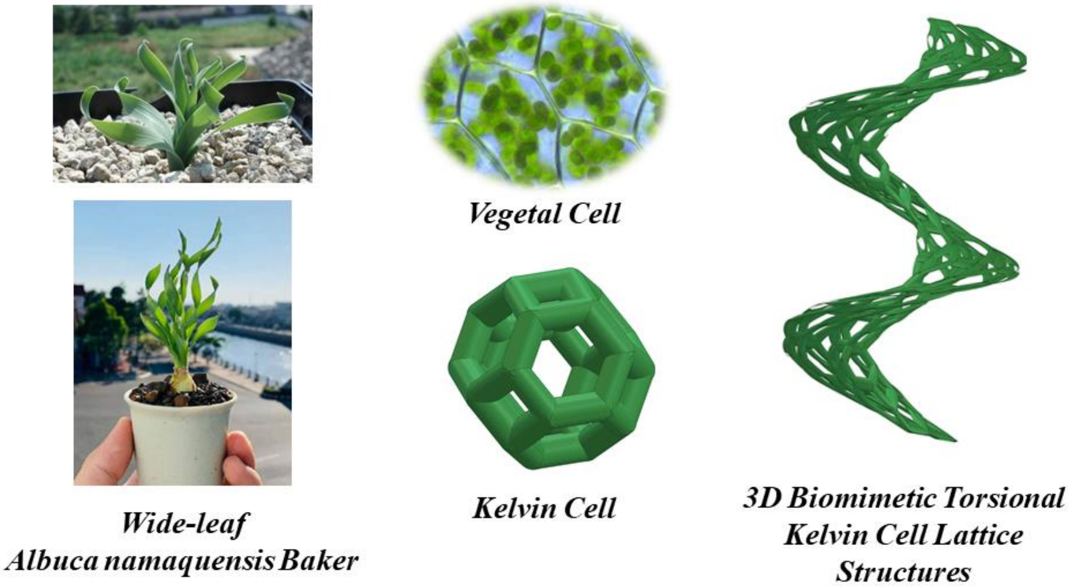

Figure S1 Design ideals of the Wide-leaf *Albuca namaquensis Baker*-like biomimetic structures.

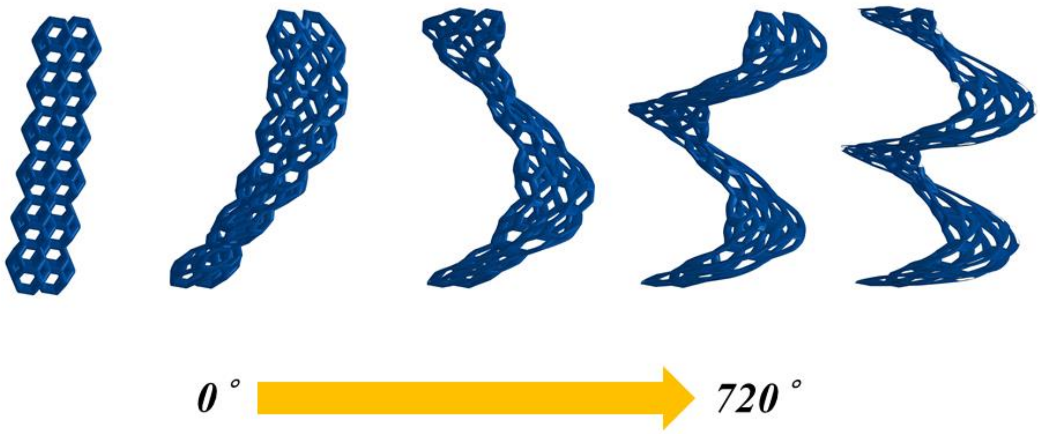

Figure S2 3D models of the biomimetic torsional Kelvin cell lattice structures with different twist angles.

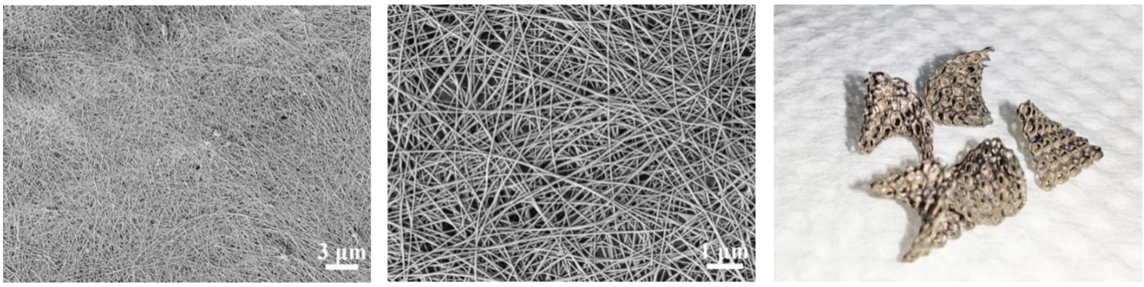

Figure S3 3D-printed biomimetic metallic SiOC ceramic torsional Kelvin cell lattices.

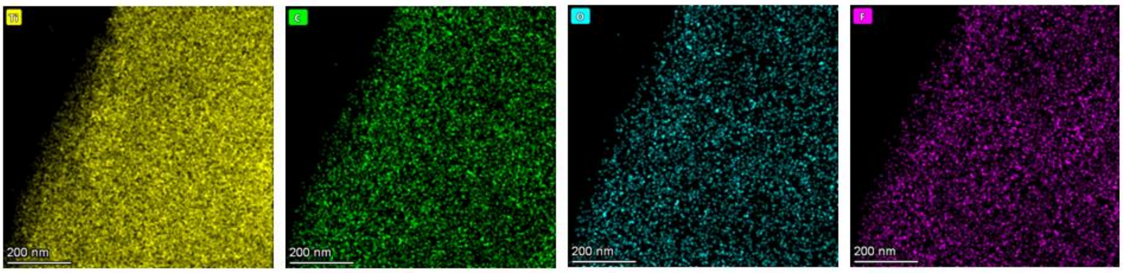

Figure S4 EDS elemental maps of Ti, C, O, and F for GQDs/MXene.

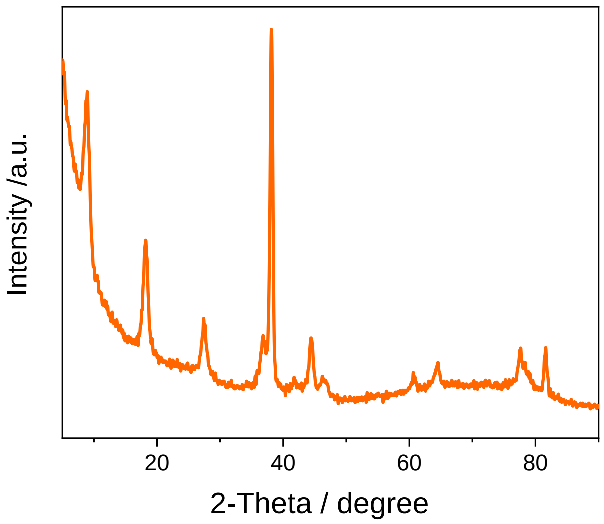

Figure S5 XRD pattern of Ti_3AlC_2.

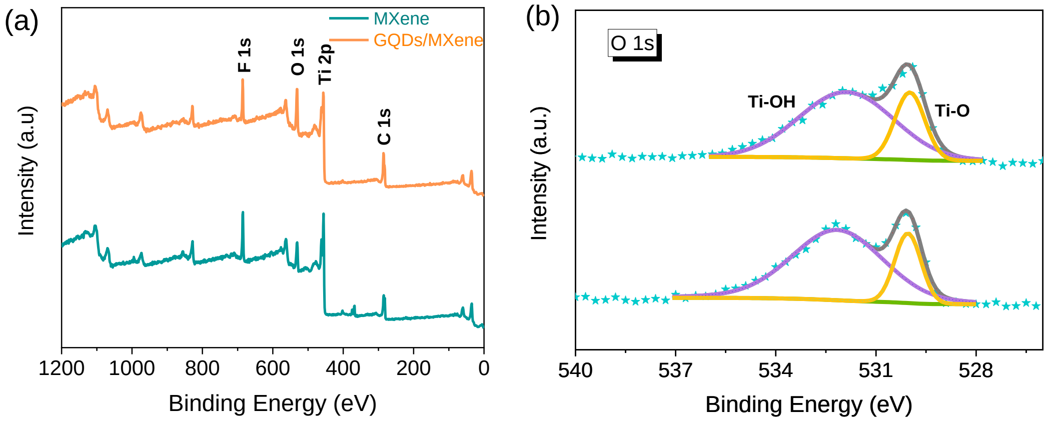

Figure S6 a) XPS survey and b) high-resolution O 1s spectra for MXene and GQDs/MXene.

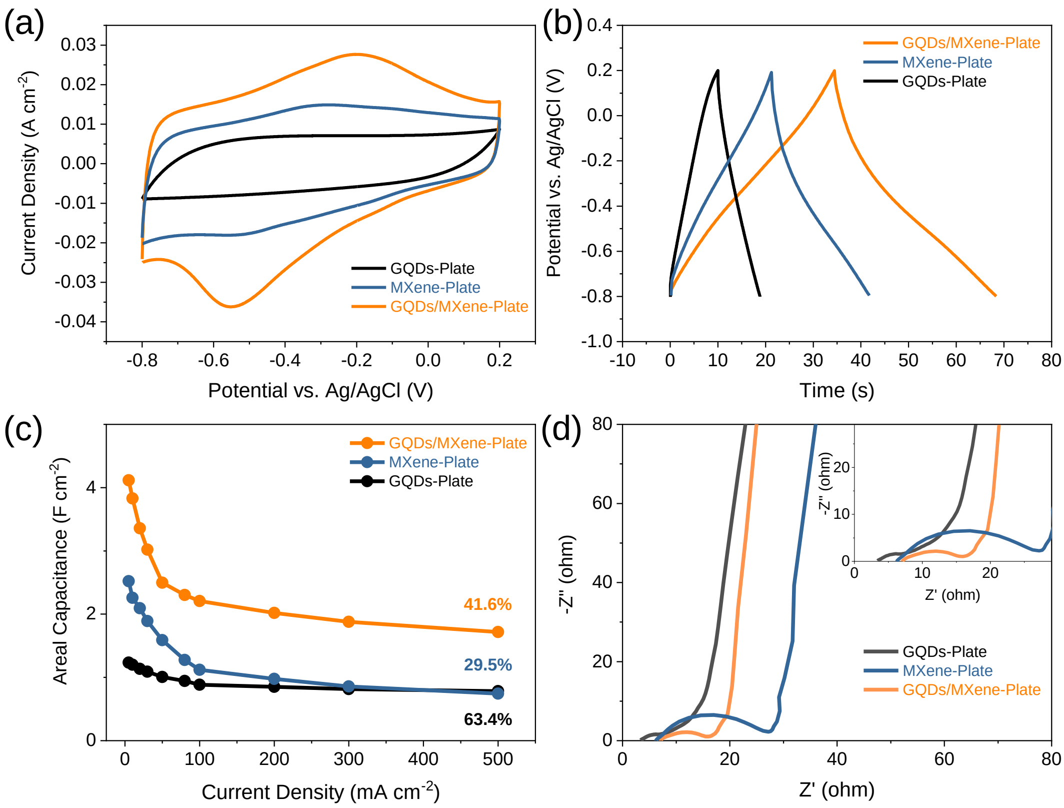

Figure S7 Electrochemical performance of GQDs-Plate, MXene-Plate and GQDs/MXene-Plate electrodes. a) CV curves at 5 mV s^{-1}, b) GCD curves at 0.1 A cm^{-2}, c) rate capability, and d) Nyquist plots.

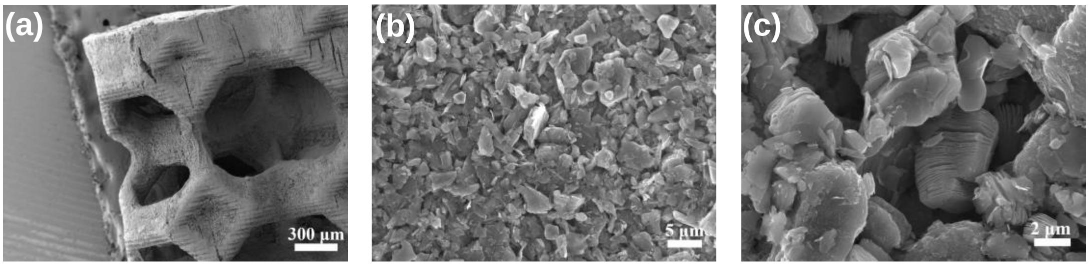

Figure S8 SEM images of 3D-printed Kelvin cell lattices after GQDs/MXene coating.

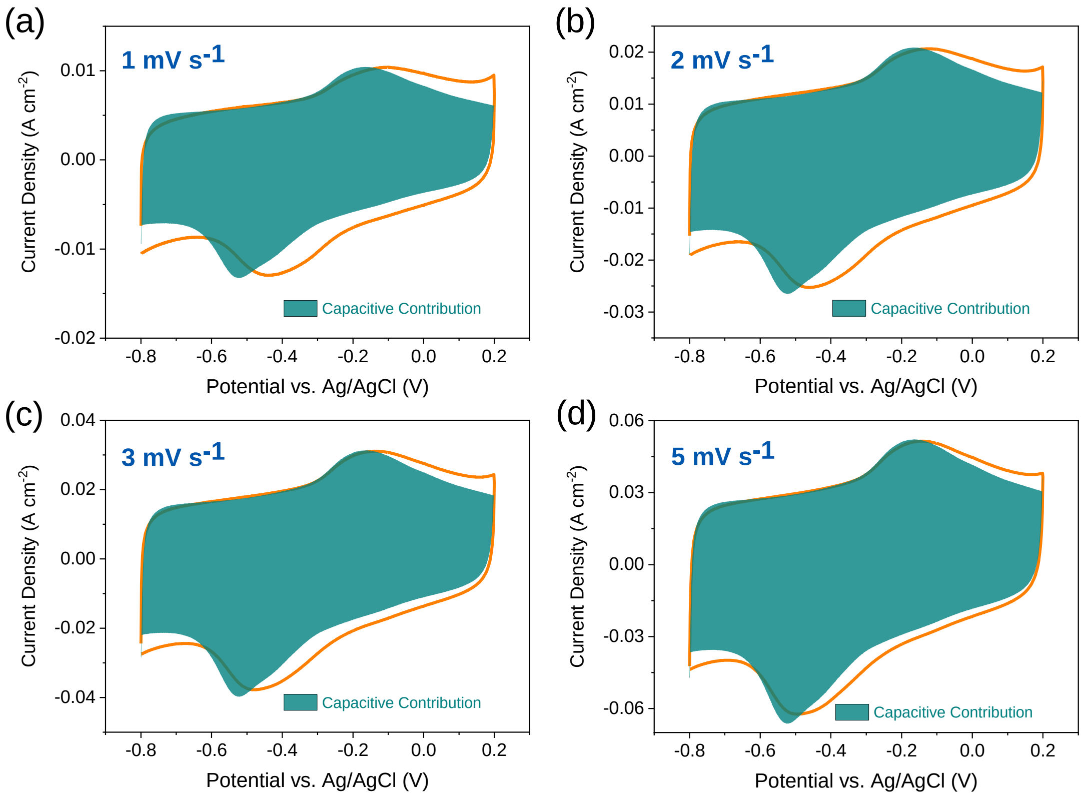

Figure S9 Separation of the capacitive and diffusion currents in the GQDs/MXene-720° electrode at a) 1 mV s^{-1}, b) 2 mV s^{-1}, c) 3 mV s^{-1} and d) 5 mV s^{-1}, depicting the capacitive contribution indicated as masked region.

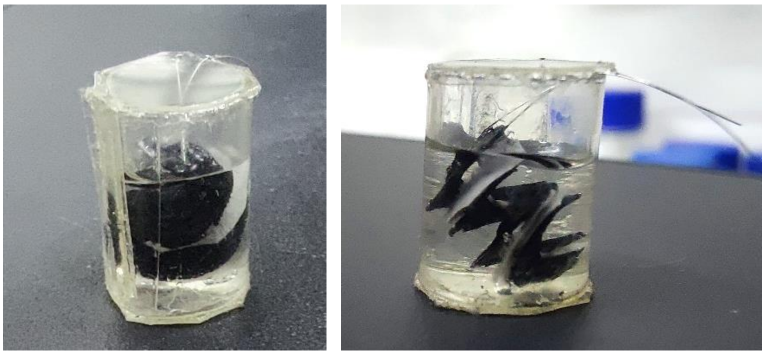

Figure S10 Device photos of the as-assembled GQDs/MXene-720° supercapacitor.

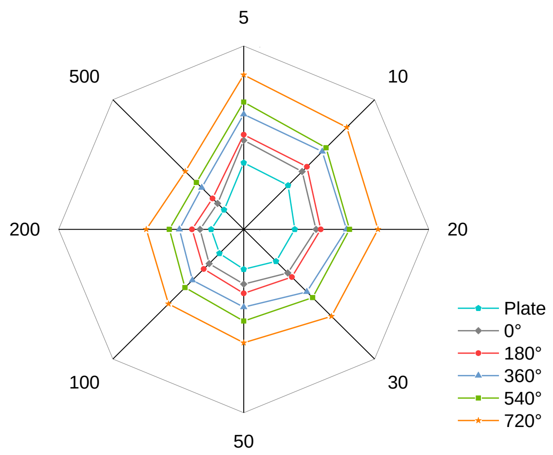

Figure S11 Rate capability of the GQDs/MXene supercapacitors with different twist angles in the dark.

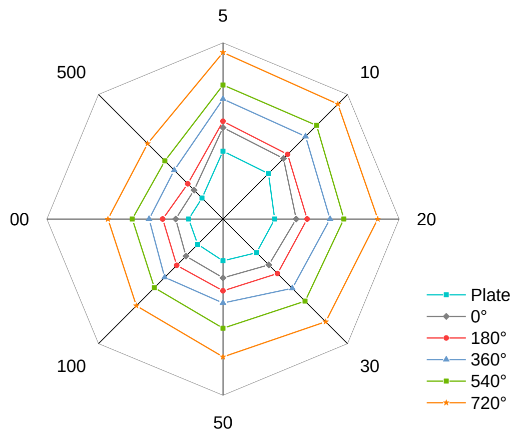

Figure S12 Rate capability of the GQDs/MXene supercapacitors with different twist angles under 1 solar illumination.

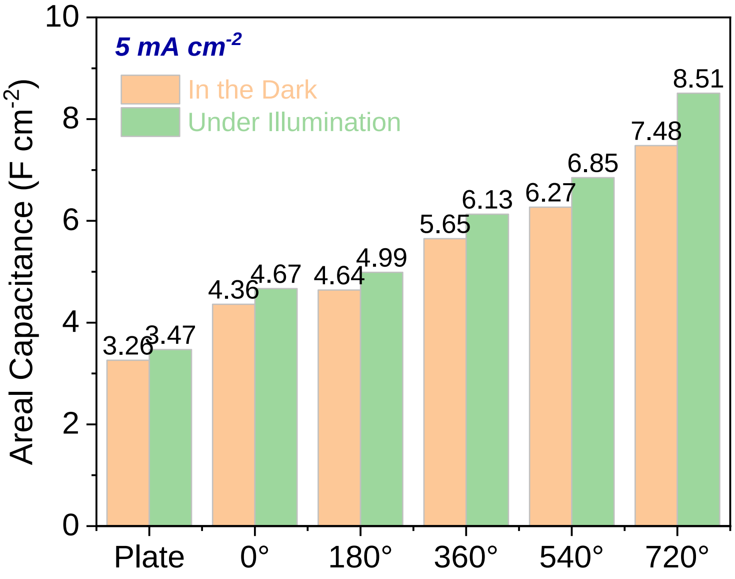

Figure S13 C_A values of the GQDs/MXene supercapacitors with different twist angles in the dark and under 1 solar illumination at 5 mA cm^{-2}.

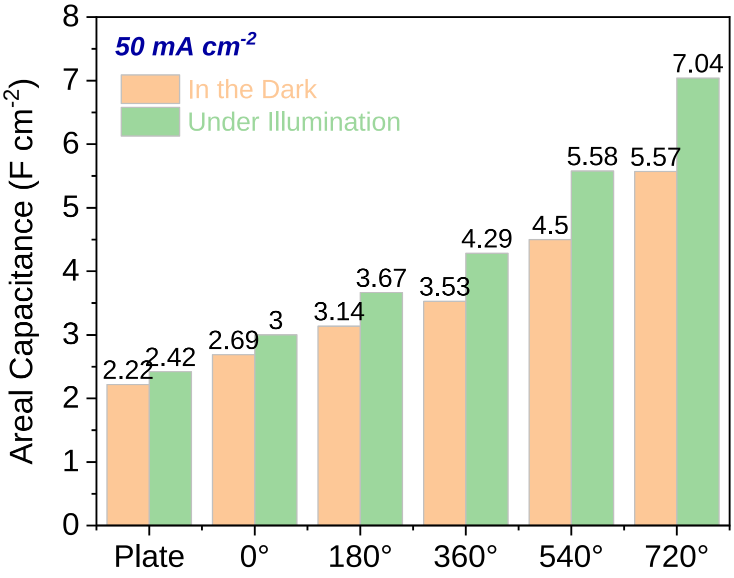

Figure S14 C_A values of the GQDs/MXene supercapacitors with different twist angles in the dark and under 1 solar illumination at 50 mA cm^{-2}.

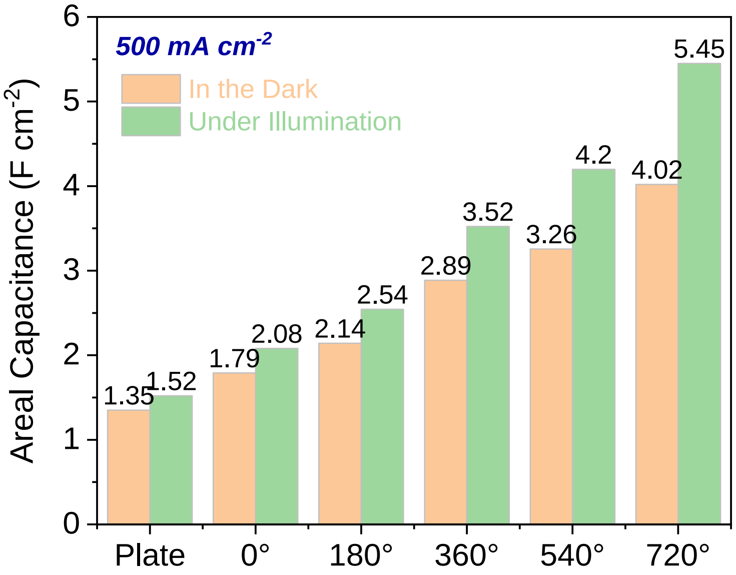

Figure S15 C_A values of the GQDs/MXene supercapacitors with different twist angles in the dark and under 1 solar illumination at 500 mA cm^{-2}.
